# Testing self-supportive strategies to regulate autonomy and motivation

**DOI:** 10.1371/journal.pone.0311264

**Published:** 2024-10-28

**Authors:** Barbara Flunger, Lau Lilleholt, Robert Böhm, Anouk Verdonschot, Tamara van Gog, Ingo Zettler

**Affiliations:** 1 Department of Education, Utrecht University, Utrecht, The Netherlands; 2 Centre for the Experimental-Philosophical Study of Discrimination, Aarhus University, Aarhus, Denmark; 3 Department of Psychology, University of Copenhagen, Copenhagen, Denmark; 4 Copenhagen Center for Social Data Science (SODAS), University of Copenhagen, Copenhagen, Denmark; 5 Faculty of Psychology, University of Vienna, Vienna, Austria; St John’s University, UNITED STATES OF AMERICA

## Abstract

People regularly encounter situations in which they have to engage in tasks they find boring or irrelevant, in which case their autonomy—the need to act in ways that are meaningful for oneself—is impeded. When there is no motivational support available, individuals need to find ways to overcome their motivational barriers by themselves. Applying autonomy-regulation strategies may be effective for increasing autonomy and particularly the more adaptive types of motivation (i.e., intrinsic and internalized). Testing this idea, we investigated whether individuals apply self-supportive strategies to boost their feelings of autonomy and motivation in two studies via cross-sectional survey samples (overall *N* = 2,344). We analyzed how autonomy regulation strategies were associated with theoretically related variables via Structural Equation Modeling (SEM). In Study 1, the self-reported use of autonomy regulation strategies was strongly positively associated with intrinsic and internalized types of motivation, weakly positively correlated with introjected avoidance motivation, and not associated with external motivation. In Study 2, we introduced two dilemmas concerning motivational problems individuals face when engaging in tasks and being assigned a task, respectively. Results indicate that individuals report using general strategies of autonomy regulation to achieve fulfillment of autonomy and intrinsic motivation, next to more specific autonomy regulation strategies regarding specific dilemmas. Our results show that it would be worthwhile to investigate if stimulating individuals to use autonomy regulation strategies would positively affect their motivation (e.g., in job or study contexts), and that in doing so, it is important to consider both specific context effects on autonomy regulation as well as individual preferences for general strategies used to regulate one’s autonomy.

## Introduction

According to Self-Determination Theory (SDT), autonomy is a basic psychological need [1]. Individuals feel autonomy when they sense that they can act in ways that align with their values, goals, and interests [2]. Yet, individuals often encounter situations that undermine their need for autonomy [1] because they have to perform tasks that they find boring or irrelevant; consequently, they struggle to stay motivated [3].

SDT [1] claims that all motivated actions stem from extrinsic or intrinsic motives (e.g., [4]). Extrinsically or intrinsically motivated actions can be categorized according to the “relative autonomy continuum” [5], which captures whether a behavior is undertaken with a sense of lack of or low autonomy (external motivation), some autonomy (e.g., introjected motivation) or complete autonomy (intrinsic motivation). If behavior is being controlled or driven by external contingencies it is categorized as external motivation; if external causes have been partly internalized it is understood as introjected motivation. By comparison, extrinsically motivated behaviors can also become internalized to self-relevant (identified), or fully accepted (integrated) behaviors, depending on the degree of autonomy perceived (e.g., [6]). When individuals feel that the reason for performing a task is completely self-determined, they perceive the task as inherently interesting and enjoyable (i.e., they are intrinsically motivated). Given that intrinsic motivation has been shown to be more beneficial than extrinsic types of motivation for several important life outcomes, such as well-being (e.g., [7, 8]), and that identified motivation is a key predictor of persistence [7] and job performance [8], it is important to identify strategies for supporting intrinsic motivation and identified motivation.

Many empirical studies have shown that autonomy support can effectively boost intrinsic and identified motivation (for a meta-analysis see [9]) when applied within hierarchical structures (“in the relations of authorities with subordinates,” [10], p. 176). It is an empirical question whether autonomy support is also effective when used “in the relationship of a person with themselves” ([10], p. 176).

Enhancing interest and self-relevance are well-known motivational regulation strategies that can enable individuals to increase their effort (e.g., [11, 12]) or self-efficacy beliefs [13]), amongst other positively-valenced outcomes. Importantly, though, two research gaps can be identified in the existing literature on motivation regulation. First, the existing literature does not yet tell us which type of motivation is promoted or maintained when certain motivation regulation strategies are applied. Second, the associations of motivation regulation strategies with introjected or identified motivation have not yet been investigated in detail. Therefore, it is an open question how individuals’ autonomy regulation is associated with the quality of their motivation (i.e., the degree to which an individual’s motivation is intrinsic versus extrinsic). Tackling these gaps, we study the associations of self-supportive strategies to enhance autonomy with satisfaction of the need for autonomy, intrinsic motivation, and distinct types of extrinsic motivation.

### The autonomy continuum of motivation

According to SDT (e.g., [1]), motivation amounts to the question of *why* one executes certain activities. Different regulation styles are claimed to direct specific actions, which can be characterized by the relative degree of autonomy achieved through action and qualitatively different intrinsic and extrinsic reasons for acting. Intrinsic motivation is characterized by high autonomy because the activity itself is deeply satisfying [14]. External motivation implies low autonomy, because behaviors are not controlled by one’s interests or values, but exclusively by extrinsic reasons. Extrinsically motivated behaviors can become internalized to self-relevant (identified) or fully accepted (integrated) behaviors, depending on the degree to which they enable the expression of personal values (e.g., [6]) and grant autonomy. Identified motivation means that individuals can identify with the value of the activity and embrace the reasons for performing it (e.g., [14, 15]). Thus, individuals have internalized the value of the behavior and as a result, they feel some ownership and choice (i.e., autonomy). If an extrinsic reward is experienced to improve self-evaluation or self-esteem, this results in (partially internalized) introjected motivation (e.g., [6]). Introjected motivation corresponds with low autonomy because behaviors result from internal pressures, such as concerns about guilt or self-worth. Recent research differentiates between introjected approach motivation ([16], also labeled as positive introjection, [6]) which implies task engagement as a means to attain higher self-worth, and introjected avoidance motivation (or negative introjection, see [6]), which reflects task engagement as a means to avoid losses in self-esteem [6, 16]. Because external and introjected motivation are primarily driven by external forces, they have been termed controlled motivation (e.g., [6]). Identified, integrated, and intrinsic motivation, by contrast, are characterized by a greater level of autonomy. Consequently, these types of motivations have become understood as autonomous motivation (e.g., [6]).

Motivation is a key factor underlying important life outcomes. Generally, intrinsic and autonomously internalized forms of motivation, i.e., integrated and identified motivation, can be considered to be more beneficial. In detail, intrinsic, integrated, and identified motivation have been found to be associated with positively-valenced academic and health outcomes, such as engagement, psychological health, subjective well-being, and hope, in different samples ranging from undergraduate students to adults across the general population (for recent reviews see [7, 8]). In contrast, controlled types of motivation have been found to be associated with negatively-valenced outcomes, such as burnout and depressive symptoms (e.g., [17]). In the work context, studies have found that intrinsic motivation can have a positive influence on work performance, positive work-related attitudes, job satisfaction, and well-being [18]. Low intrinsic motivation can be problematic because it increases the likelihood that employees engage in counterproductive behavior and will contribute less to the goals of an organization. Extrinsic types of motivation can also increase other negatively connoted outcomes (such as turnover intention and burnout [19]) and reduce performance [20].

Therefore, it is desirable to boost individuals’ intrinsic motivation. Because the relative level of autonomy attained is a defining feature of the distinct motivation types, autonomy can be understood as the key to rendering extrinsic motivation into identified or intrinsic motivation.

### Autonomy crafting and regulation

In SDT research, perceived need satisfaction has predominantly served as a proxy for studying basic psychological needs. However, Ryan and Deci [4] claim that individuals “may differ in terms of how subjectively salient these needs are or how centrally the needs are represented in their personal goals and lifestyles, and these individual differences might affect need satisfaction” (p. 242). Thus, autonomy need satisfaction can be accomplished through personal strivings for autonomy. Consequently, monitoring one’s feelings and reactions has been forwarded as an aspect of autonomy, next to authorship, self-congruence, and susceptibility to control [21].

A body of empirical research has provided evidence on how autonomy need satisfaction can be supported via contextual strategies. For instance, one effective autonomy-supportive strategy can be to stimulate people’s interests by offering tasks that match their interests, by providing rationales (e.g., on why it is relevant to perform a certain task), or by providing choices between activities (e.g., [22]).

Importantly, individuals also apply these strategies themselves to increase their autonomy and ultimately render a context or a task more motivating [10, 23, 24]. For example, creating choices through changing activities is a self-regulatory strategy individuals report using spontaneously when facing challenges in everyday activities [24]. These strategies resemble effective motivational regulation strategies highlighted in educational research (self-enhancement of personal relevance or interest) which can buffer against motivational declines, (e.g., [25]). Rooted in distinct theoretical frameworks (expectancy-value theory, achievement goal theory, and frameworks on self-regulation and self-regulated learning, see e.g., [26]), motivation regulation refers to the active monitoring of the willingness to engage (e.g., [3]), including “awareness, and regulation of one’s thinking” (defined as metacognition, [27], p. 299) and monitoring one’s own motivation (e.g., [28]).

Based on SDT, Legault et al. [29] proposed the concepts of assisted and asserted autonomy which describe individuals’ active strivings for greater autonomy need satisfaction. It needs to be noted that SDT separates autonomy (willingly enacting and self-endorsing actions, [30]) from independence (“not relying on others for support, help, or supplies,” [30], p. 98). Strivings for independence might be expressed through asserted autonomy strivings [29], emphasizing “the personal claiming of autonomy through one’s independence” [31, p. 901], also when engaging with other people. Asserted autonomy strivings, thus, characterize the tendency to push for autonomy. By comparison, assisted autonomy strivings indicate a preference for ease and harmony in interpersonal interactions when striving for autonomy, likely due to prior experiences of high need support, e.g., in close relationships [31].

Recently, the concept of need crafting [32] was introduced which, similar to the concept of job crafting (e.g., [33]), highlights the idea that individuals actively monitor their need satisfaction and take actions to maintain or increase it. More specifically, job crafting refers to proactive behavior by employees to modify their jobs and/or perceptions of their jobs, to increase meaning, engagement, and job satisfaction [33]. Need crafting has a close correspondence with motivation regulation because it can reflect individuals’ “attempts to exert control over their self-determination, especially when faced with [e.g.,] academic challenges that may undermine it” [34, p. 3].

Autonomy crafting involves metacognitive monitoring of autonomy-based experiences in specific contexts, and identifying possibilities to render them more autonomy-supportive for oneself. Specifically, individuals can strive to create autonomy-based experiences to satisfy their need for autonomy by themselves: Individuals might actively engage in the pursuit of their need for autonomy by intentionally seeking out and creating experiences that are centered around autonomy, a process that can be termed autonomy regulation, aimed to induce experiences that allow the enactment of the self.

On the one hand, need crafting may be a response to a perceived lack of need satisfaction. Namely, a psychological need can function as a motive and “elicit goal-oriented behavior designed to satisfy it” [35, p. 1468]. On the other hand, individuals with high autonomy need satisfaction might be more likely to craft their autonomy, because they value the experience of autonomy. Namely, autonomy need satisfaction can lead to a general valuing of autonomy [36, 37]. Individuals in this case have learned that the fulfillment of autonomy is linked to positive outcomes [35]. As a result, need crafting might also resemble an intentional strategy of striving for need satisfaction which does not necessarily stem from a perceived lack of need satisfaction but an individual’s tendency to create a motivationally supportive environment for oneself.

In sum, individuals can proactively regulate their motivation and basic psychological needs with self-regulatory strategies. Research on self-regulation and motivation regulation mostly focused on self-motivating strategies aimed at changing one’s motivation when facing a motivational problem but these strategies might also affect one’s autonomy need satisfaction. Autonomy regulation might reflect a self-supportive style (i.e., a general striving) to generally aim for autonomy need satisfaction, e.g., aiming to design a self-determined life and to make autonomous choices (e.g., asserted autonomy, [29]). Autonomy regulation strategies might thus be highly correlated with autonomy need satisfaction. For example, Legault et al. [29] found positive correlations between assisted and asserted autonomy strivings and different measures of autonomy need satisfaction (see also [31]). So far, however, there have been no studies examining how autonomy need satisfaction and further motivation regulation strategies are related.

### Interpersonal and intrapersonal autonomy regulation

Autonomy-regulating strategies can be implemented to induce greater need satisfaction for oneself and regulate the distinct subtypes of the motivation continuum, particularly the types of autonomous motivation (e.g., [38]). Moreover, autonomy regulation can be applied both as an interpersonal strategy, to render interactions with others more need-satisfying and motivating, and as an intrapersonal strategy; in this case, to regulate one’s autonomy need satisfaction as well as the interpretations and behaviors in a situation.

### Interpersonal autonomy regulation: Agentic engagement

Interpersonal autonomy regulation might take the form of “agentic engagement” [39], which refers to the proactive and constructive actions individuals can apply with the aim of rendering a context more autonomy-supportive and fostering internalized forms of extrinsic motivation within a given context (e.g., [40]). Agentic engagement has mainly been studied in educational research and refers to a “student’s constructive contribution into the flow of instruction they receive” [40, p. 97]. However, agentic engagement can be applied in a variety of contexts, through making use of autonomy-supportive strategies for oneself, e.g., informing about interests, preferences, and needs in a specific situation. A large body of research has demonstrated that autonomy-supportive strategies, such as providing choices and rationales or stimulating interest, can boost intrinsic motivation in different life domains (e.g., [41, 42]; for a review in the educational domain, see [22]; for a meta-analysis in the workplace domain, see [43]). Yet, individuals can also use these strategies in interpersonal interactions to create more autonomy-supportive environments for themselves. Individuals can ask questions, express preferences, opinions, and interests, and can propose alternative approaches, to create a more autonomy-supportive environment, such as better access to interesting and personally valued activities [23]. So far, agentic engagement has mainly been treated as a global construct and has typically been assessed with five items reflecting different autonomy-oriented strategies (such as “I ask questions” or “I express my preferences and opinions,” [23]).

### Intrapersonal autonomy regulation: Motivation regulation

Individuals can use motivational regulation strategies once they realize that they have a motivational problem, and proactively strive to maintain their own motivation [44]. Eight strategies for motivation regulation have been recognized (e.g., [11]): Self-consequating with rewards (e.g., for task completion), performance-approach self-talk (e.g., focusing on how important it is to obtain good grades), performance-avoidance self-talk (e.g., “I have to push me more if I do not want to make a fool of myself,” [11], p. 626), mastery self-talk (e.g., telling oneself to learn as much as possible for oneself), enhancement of situational interest (e.g., rendering the task into a game), enhancement of personal significance (e.g., identifying why task engagement could be personally meaningful), proximal goal setting (e.g., splitting tasks in sub-steps), and environmental control (e.g., reorganizing the learning environment before starting to work on a task).

When evaluating conceptual similarities between the concepts of autonomy support and motivation regulation, it is noteworthy that the strategy *enhancement of situational interest* has close conceptual similarities with the autonomy-supportive strategies of creating choices (e.g., through varying the procedure) and stimulating interest (rendering the task into a game). Providing rationales is similar to the strategy *enhancement of personal significance*: Instead of being told why a specific rule is important to be followed in a specific context or for task completion, an individual can also generate self-relevance. Thus, when aiming to examine strategies directed at increasing feelings of autonomy, the motivational regulation strategies *enhancement of situational interest*, *enhancement of personal significance*, and *creation of choices* have the closest match with autonomy (support).

### Associations with intrinsic and extrinsic types of motivation

Research on the associations of motivation regulation with outcomes mostly focused on the specific construct the strategies were supposed to regulate, such as effort and persistence [11, 45], achievement goals [3, 13], self-efficacy [13], subjective task value beliefs [44], and academic achievement [45].

Only recently researchers have started to investigate how motivation regulation is associated with extrinsic and intrinsic types of motivation. Self-enhancing interest can be assumed to foster interest, the positive feelings and valences attached to a topic or activity [46, 47]. Interest has conceptual overlaps with intrinsic motivation [48], concerning the positive feelings [47] and valences attached to a topic or activity [46]. Therefore, one can expect that individuals maintain their intrinsic motivation through self-regulating their interest. Similarly, individuals can foster self-relevance of materials or activities, through highlighting how it relates to their daily life or future life plans. In doing so, they can increase the personal importance, which can be claimed to be a facet of identified motivation [49].

In a sample of 193 university students over one semester, Garn and Morin [34], for instance, found consistently positive correlations between global motivation regulation and autonomous motivation (a composite score of intrinsic and identified motivation). Moreover, in a sample of 716 Russian university students, positive associations between intrinsic motivation and self-enhancing interest and personal relevance, and positive associations between introjected motivation and the strategy of self-enhancing interest were found [50].

In experimental research with a sample of 90 undergraduate students in the US, Sansone and colleagues [51] showed that applying interest-enhancement strategies, through turning the task into a game, can induce higher intrinsic motivation (i.e., enjoyment, for more information, see Study 1) and change a boring into an interesting task. Moreover, the outcomes considered in earlier research have (some) conceptual similarities with extrinsic and intrinsic forms of motivation. Concerning extrinsic types of motivation, for example, small, negative correlations between overall motivation regulation and performance-avoidance achievement goals (which can be understood to have similarities with introjected avoidance motivation in its objective to avoid losses to self-esteem) were found in a study with 396 university students in the US [12]. With regard to autonomous types of motivation, a study with 215 German university students showed that subjective task value beliefs, which partly overlap conceptually with identified motivation (e.g., [49]), are positively affected by motivation regulation [52]. In contrast, Kim and coauthors [13] did not find an association between motivation regulation and perceived usefulness and importance of students’ current course materials.

Concerning agentic engagement, a medium-to-large, positive correlation (*r* = .44) between autonomous motivation and agentic engagement, as well as a small negative correlation (*r* = -.14) between controlled motivation (a composite between introjected and external motivation) and agentic engagement was found in a sample of 248 Korean college students (Study 2, [23]).

The motivational regulation strategies, and in particular the strategies of enhancement of interest and self-relevance, have been found to be substantially correlated (e.g., [45]). This can cause problems of multicollinearity if the motivational regulation strategies are modeled as predictors of outcomes: Assessing the unique predictive effects of the highly correlated strategies on outcomes might lead to biased estimates and suppression effects [53]. In order to circumvent this problem, several empirical studies have relied on a global, higher-order factor [13, 32, 45].

Based on prior research grounded in different theoretical frameworks, it can be expected that autonomy regulation has stronger associations with intrinsic and identified motivation than with introjected avoidance motivation and external motivation (e.g., [6]). In prior empirical research, motivation has often been modeled as a global autonomous motivation factor (a composite variable of intrinsic, integrated, and identified motivation, e.g., [6]) and global controlled motivation factor (a composite variable of introjected and external motivation, e.g., [15, 54–56]), or an overall factor of self-determination, e.g., [57]). However, the examination of the association between autonomy regulation and the specific types of extrinsic and internalized motivation is understudied, emphasizing the necessity to investigate these associations with different types of motivation separately.

### Purpose of investigation and overview of the two studies

Several lines of research focus on individuals’ proactive efforts to regulate their motivation and basic psychological needs: These research traditions either focused on self-motivating strategies (research on motivation regulation, e.g., [25, 34]) or need crafting [32]. Similar self-motivating and autonomy-regulating strategies can be identified based on different theoretical frameworks (SDT and self-regulation), indicating conceptual overlap. Autonomy regulation can be applied both as an interpersonal strategy in interactions (which has been highlighted in research on agentic engagement, [39]) and as an intrapersonal strategy to boost own autonomy need satisfaction and motivation.

It can be assumed that strategies of autonomy regulation can effectively enhance autonomy need satisfaction and adaptive motivation. Yet, it is unclear whether self-supportive strategies to alter types of motivation, strategies to induce greater autonomy need satisfaction for oneself, as well as intrapersonal and interpersonal autonomy regulation (agentic engagement) can be empirically distinguished. A systematic differentiation between these concepts is missing, so far. Therefore, the purpose of the present studies was to investigate whether different conceptualizations of autonomy regulation hold empirically. The second objective was to investigate how autonomy regulation strategies are associated with perceived autonomy need satisfaction, intrinsic motivation, and distinct types of extrinsic motivation.

In Study 1, we first examine whether the strategies individuals use to self-generate choices, interest, and self-relevance for oneself (intrapersonal strategies), can be differentiated empirically from agentic engagement (interpersonal strategy). Second, we investigate how these autonomy regulation strategies are associated with perceived autonomy, intrinsic motivation, and distinct types of extrinsic motivation.

In Study 2, we investigate how strategies to self-generate choices, interest, and self-relevance are being used for both intra- and interpersonal autonomy regulation, and how they are associated with perceived autonomy and intrinsic motivation. In addition, to be able to compare self-supportive strategies to regulate motivation versus self-supportive strategies that aim to increase autonomy-based experiences, we introduced a measure of general autonomy strivings (which we assumed to correlate highly with autonomy need satisfaction but to be a conceptually distinct construct).

The hypotheses, design, and analyses were preregistered (Study 1: https://aspredicted.org/38sf-pnjp.pdf; Study 2: https://aspredicted.org/hf2p-wnvm.pdf). All participants were 18 years or older and provided written informed consent. We report how we determined our sample size and all measures used in this research. Note that there were additional variables in the survey that were not relevant for the present research and were therefore not included in the preregistration. We did not exclude any data. All preregistered analyses, outputs, and research materials are available on the Open Science Framework (OSF, https://osf.io/96dm7).

## Study 1

The aim of Study 1 was to examine a measure of autonomy regulation. First, we assessed whether the theoretical differentiation of the four strategies (creating choices, enhancing interest, fostering self-relevance, agentic engagement) also holds empirically. We expected to be able to differentiate these four autonomy regulation strategies and that these four factors load on a global, higher-order factor [32, 45].

**Fig 1:**
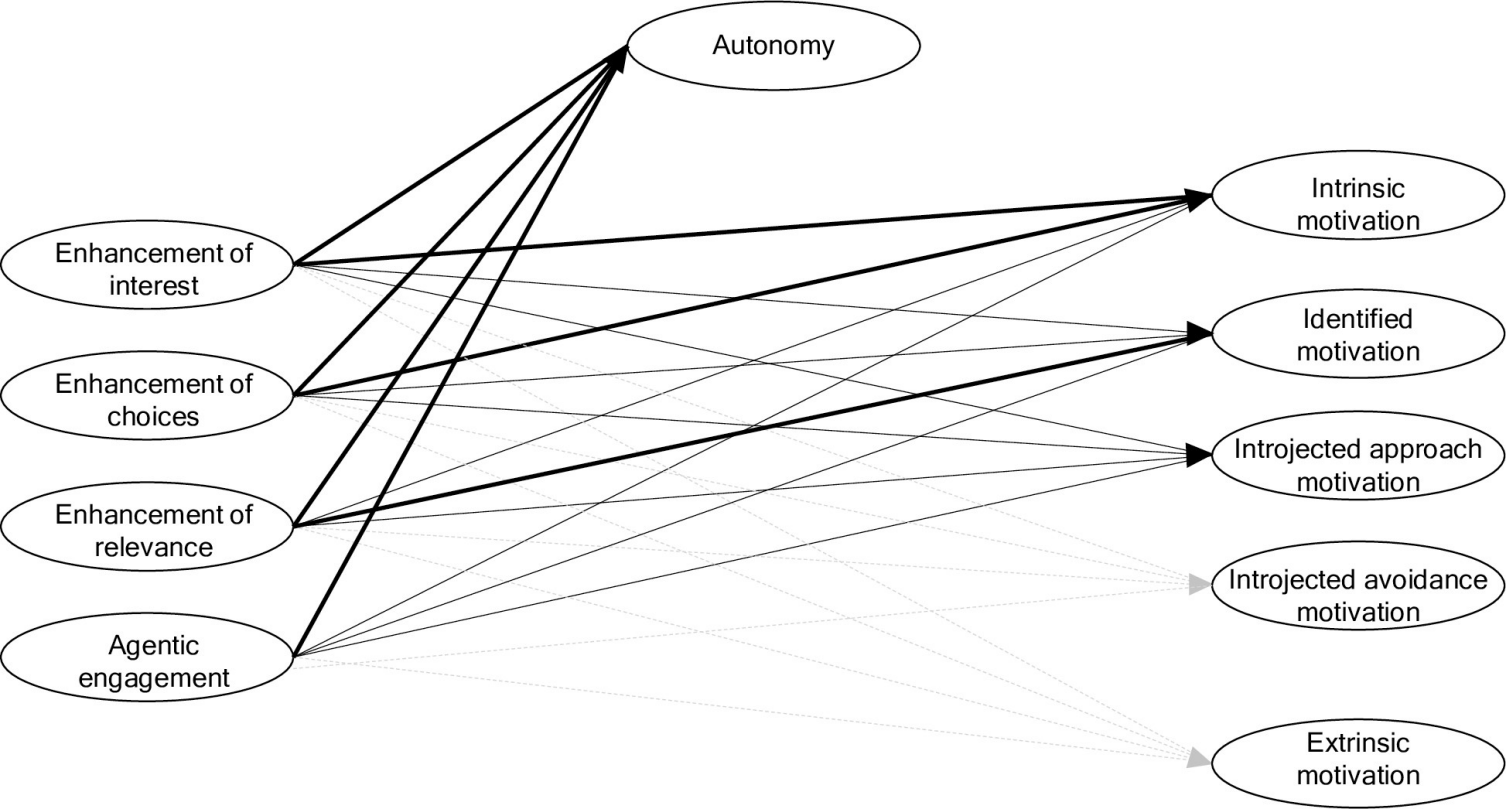
Associations between the autonomy regulation strategies, autonomy need satisfaction and types of motivation. Black lines signalize positive associations, dashed lines refer to modest associations. Strength of relationship is signalized through line thickness.

Second, we aimed to investigate how autonomy regulation was associated with theoretically related variables, namely, autonomy, intrinsic, identified, introjected approach, and avoidance motivation, as well as external motivation. We assessed whether autonomy need satisfaction and autonomy regulation were distinct constructs. We expected that autonomy regulation would be positively correlated with autonomy, intrinsic, identified motivation, and introjected approach motivation. By comparison, we expected that autonomy regulation was only modestly positively associated with introjected avoidance and fully extrinsic motivation (see Fig 1).

### Method

#### Participants and procedure

Data were collected via a repeated cross-sectional survey sample. More precisely, following data protection, in weeks 29 and 31, 2020, 14,000 individuals from a (concerning age and gender) representative sample of Danish adult citizens were invited to participate in a survey on perceptions and behaviors related to COVID-19 (data collection was open to all invited participants until Sunday of the respective week [58]). From all individuals responding to the invitations and providing active informed consent, 1,675 participants (43.10% males, 53.3% females, 0.30% Other, *M*_*age*_ = 57.50, *SD* = 15.82 years) completed the surveys and were included in Study 1.

#### Measures

All scales used herein were measured with a 5-point Likert-type scale ranging from 1 = *strongly disagree* to 5 = *strongly agree*. The items were either taken from the literature or developed based on the literature, and then translated from English into Danish (via student assistants being blind to the purpose of this study, and then finally adapted and approved by the 2^nd^ author, being native in Danish). All internal consistencies are reported in Table 1.

**Table 1:** Descriptive statistics, internal consistencies (Cronbach’s alpha), and intercorrelations of all variables (Study 1).

	*M*	*SD*	α	(2)	(3)	(4)	(5)	(6)	(7)	(8)	(9)	(10)	(11)
Motivation													
Intrinsic motivation	3.42	.87	.82	.56***	.64***	.09**	-.32***	.38***	.29***	.44***	.39***	.40***	.44***
Identified motivation	4.26	.61	.82		.83***	.17***	.00***	.51***	.42***	.41***	.47***	.55***	.51***
Introjected approach motivation	3.44	.55	.60			.43***	.15***	.43***	.37***	.40***	.44***	.51***	.48***
Introjected avoidance motivation	3.06	.98	.76				.72***	.10***	.06***	.15***	.11***	.17***	.15***
Extrinsic motivation	1.79	.45	.59					-.03***	-.01***	-.00***	.02***	.02***	.02***
Autonomy need satisfaction													
Autonomy	3.94	.45	.71						.72	.71***	.76***	.87***	.83***
Autonomy regulation													
Agentic engagement	3.74	.58	.71							.64***	.62***	.80***	.73***
Enhancement of interest	3.90	.58	.69								.90***	.86***	
Enhancement of relevance	4.15	.53	.67									.86***	
(10) Enhancement of choices	3.92	.45	.61										
(11) Higher-order AR	-	-	-										

*Note*. Sample size varied for the distinct scales from *N* = 1,656 (motivation) to *N* = 1,675 (autonomy regulation, autonomy). AR = Higher-order factor autonomy regulation.

*p < .05.

**p < .01.

***p < .001.

#### Agentic engagement

Agentic engagement was assessed with a scale adapted from the five-item Agentic Engagement Scale [23]. We used the following four items: “I tell others what I like and what I don’t like,” “I express my preferences and opinions,” “I offer suggestions about how to improve something if I do not like it,” and “When I need something, I’ll ask for it.”

#### Autonomy regulation

The self-regulation of autonomy was assessed with a focus on the use of three self-supportive strategies: stimulating one’s own interest, providing rationales, and providing choices for oneself [22]. Three items were created for each construct, based on scales by Schwinger et al. [11]. All items are listed in Table 2.

**Table 2:** Standardized factor loadings 4-factor CFA (Study 1).

		Latent factor			
Label	Item	Agentic engagement	Choice enhancement	Interest enhancement	Relevance enhancement
NRSR1	I tell others what I like and what I don’t like.	.63			
NRSR2	I express my preferences and opinions.	.72			
NRSR3	I offer suggestions about how to improve something if I do not like it.	.63			
NRSR4	When I need something, I’ll ask for it.	.52			
CC1	Whenever possible, I adjust tasks to my preferences.		.56		
CC2	I do what I can to improve my environment so that it fits me better.		.58		
CC3	I aim to achieve what I want.		.61		
IE1	I try to make whatever I do as interesting as possible.			.70	
IE3	I make myself look for ways to bring more fun to a task.			.69	
IE3	I try all kinds of different things to stimulate my interest.			.59	
RE1	I aim to create meaning for myself in the things I undertake.				.68
RE2	I look for connections between my tasks and my life as such.				.49
RE3	I consider a way to make tasks personally meaningful.				.78

*Note*. *N* = 1,675. NRSR = Need-related self-regulation (agentic engagement). CC = Creating choices. IE: Interest enhancement. RE: Relevance Enhancement. CFA = Confirmatory factor analysis. All factor loadings were statistically significant (*p* < .001). Based on statistically significant modification indices, correlated residuals were specified between two item pairs, namely, between RE2 (I look for connections between my tasks and my life as such) and IE3 (I try all kinds of different things to stimulate my interest), as well as between CC3 (I aim to achieve what I want) and NRSR4 (When I need something I’ll ask for it).

#### Motivation

Extrinsic and intrinsic forms of motivation were measured with a scale developed by Sheldon et al. [6]. Participants were first asked to consider the extent to which they take responsibility for four different life domains—self-development, health, own happiness and future, and work or studies—and then asked to select the personally most important life domain. Individuals need to navigate their goals and motivation across distinct life domains, e.g., family, work, education, or self-development (e.g., [59]). There is quite some variance concerning the extent to which individuals are invested in different life domains [59, 60] which implies that several life domains should be represented or a generic formulation should be used when measuring trait motivation, in order to capture the diversity of individual’s life-domain investments. The measure of Sheldon et al. [6], in which respondents evaluate motivational internalization referring to personal responsibility for their most important life domain, targets trait-specific motivation, which refers to motivation at a global level. More specifically, our measure reflects a general motivational orientation [61] and assesses internalization and the degree of importance of a domain through the feeling of being responsible [6]. Sheldon et al. [6] compared different stems, and the findings regarding the structure of the measure were replicated, independently of whether the introductory question “why do you take responsibility” or “why do you go to class/choose your major” was used. Therefore, the authors expect that their measure works with distinct stems (for more information see [6]). In both Study 1 and Study 2, most of our participants chose health as most important life domain, which might be due to the survey being conducted during the COVID-19 pandemic, followed by own happiness and future, self-development, and finally, work or studies.

Intrinsic motivation was measured with 3 items (“because it is fun,” “because it is a pleasure to do it,” “because it is interesting”). Identified motivation was measured with 4 items (“because I strongly value it,” “because it is personally important to me,” “because it is meaningful to me,” “because it is my personal choice to do it”). Introjected approach motivation was measured with 3 items (“because I want to prove to myself that I am capable,” “because it boosts my self-esteem,” “because I want to feel good about myself”). Introjected avoidance motivation was measured with 3 items (“because I would feel guilty if I didn’t do it,” “because I would feel ashamed if I didn’t do it,” “because I would feel like a failure if I didn’t do it”). External motivation was measured with 3 items (“because if I don’t do it, others will get mad,” “because I’ll get in trouble if I don’t do it,” “because I don’t have any choice but to do it”).

#### Autonomy need satisfaction

Perceived autonomy was measured with three items derived from a scale by Chen et al. [36]: “I feel a sense of choice and freedom in the things I undertake,” “I feel my choices express who I really am,” and “I feel that my decisions reflect what I really want”.

#### Analyses

We conducted confirmatory factor analyses (CFAs) with up to four latent factors (creating choices, enhancing interest, fostering self-relevance, agentic engagement). In addition, we tested whether the autonomy regulation factor loaded on a higher-order factor. Following for instance, Hooper and colleagues [62], we considered a set of fit criteria to evaluate model fit, using the Comparative Fit Index (CFI > .90), the Tucker Lewis Index (TLI > .90), the root mean square error of approximation (RMSEA < .08), and the standardized root mean square residual (SRMR < .08). For model selection and comparison of different models, we additionally inspected the Akaike Information Criterion (AIC) and Bayesian Information Criterion (BIC) values [63, 64]. Next to model fit, the size of factor loadings (< .50) and the explained variance of items (< .25) was evaluated.

Because we used similar items for several strategies (e.g., *I let others know which tasks I would find personally meaningful/ I would like*), associations between the indicators that were unrelated to the latent factors but related to the wording of the items were possible. We added correlated residuals between pairs of items with similar wordings (see *Notes* of Table 2) in case this improved model fit and was supported by modification indices.

In order to investigate how autonomy regulation was associated with theoretically related variables, we conducted structural equation models (SEMs) in which autonomy regulation was specified as predictor of distinct types of motivation (see https://osf.io/96dm7).

Data were analyzed using *Mplus*, version 8.9 [65].

## Results

We estimated a 4-factor CFA model, specifying four self-supportive strategies (creating choices, enhancing interest, enhancing self-relevance, agentic engagement), which yielded a good model fit (CFI = 0.971; TLI = 0.961; RMSEA = .036; SRMR = .029). All standardized factor loadings were above .50 (Table 2). Based on significant modification indices related to item covariances, we added two correlated residuals (see Table 2). Given that the correlations of the latent factors were high, we compared the 4-factor model with a 2-factor model in which all intrapersonal autonomy regulation strategies in response to the dilemma were combined (CFI = 0.951; TLI = 0.938; RMSEA = .046; SRMR = .037), and a 1-factor model in which a global factor was specified (CFI = 0.885; TLI = 0.857; RMSEA = .069; SRMR = .050). Because the AIC and BIC values of the 4-factor CFA model were relatively lower than the AIC and BIC of the 2-factor and the 1-factor model—indicating a better model fit in line with the theoretical expectations—we favored the 4-factor model (see Table 3 for an overview on all model fit indices).

**Table 3:** Model fit of CFA models (Study 1).

Model	χ^2^	SCF	df	AIC	BIC	CFI	TLI	RMSEA	SRMR
**4-factor CFA**	**182.43**	**1.33**	**57**	**51101.64**	**51356.55**	**0.971**	**0.961**	**0.036**	**0.029**
2-factor CFA	278.87	1.34	62	51223.13	51450.92	0.951	0.938	0.046	0.037
1-factor CFA	568.48	1.34	63	51609.16	51831.52	0.885	0.857	0.069	0.050

*Note*. *N* = 1,675; CFA = Confirmatory factor analysis. SCF = Scaling Correction Factor. df = degrees of freedom. AIC = Akaike information criterion. BIC = Bayesian information criterion. CFI = Comparative fit index. TLI = Tucker-Lewis index. RMSEA = Root mean square error of approximation. SRMR = Standardized root mean square residual.

The selected model is highlighted in bold.

Next, we tested a higher-order model, which had an acceptable model fit (CFI = 0.960; TLI = 0.948; RMSEA = .042; SRMR = .033). In the higher-order model, agentic engagement was specified as a separate factor, given that the standardized factor loadings concerning the higher-order factor were all .91 or higher (choice enhancement: .97; interest enhancement: .92; relevance enhancement .91), and that the correlation of global autonomy regulation with agentic engagement was lower (*r* = .73; *p* < .001). The descriptive statistics, internal consistency estimates for all scales, and intercorrelations of all variables are shown in Table 1.

We used the higher-order model in the SEM models, because as it was expected due to the high correlations between the self-regulatory strategies (e.g., [45]), the SEMs assessing unique associations of the distinct regulatory strategies yielded suppression effects. We ran SEM models to investigate the predictive effects of global autonomy regulation on autonomy, and on distinct types of motivation, alongside the predictive effects of agentic engagement. As expected, the global factor of autonomy regulation (β = .67; *p* < .001) as well as agentic engagement (β = .23; *p* < .001) had a statistically significant positive association with autonomy, explaining substantial variance in autonomy (*R*^2^ = .72). Concerning intrinsic motivation (*R*^2^ = .19), the higher-order factor of autonomy regulation had a positive association (β = .48; *p* < .001), whereas agentic engagement was not statistically significantly associated with intrinsic motivation (β = -.06; *p* = .307), which could be due to multicollinearity. The higher-order factor also had a statistically significant association with identified motivation (β = .44; *p* < .001; *R*^2^ = .26), whereas agentic engagement had no statistically significant association (β = .10; *p* = .097). Moreover, when predicting introjected approach motivation (*R*^2^ = .21), autonomy regulation showed a positive association (β = .43; *p* < .001), whereas agentic engagement was not significantly related with this outcome (β = .03; *p* = .625). By comparison, autonomy regulation had a small but positive relation with introjected avoidance motivation (β = .24; *p* < .001), whereas agentic engagement had a negative relation with introjected avoidance motivation (β = -.12; *p* = .050), although the explained variance was small (*R*^2^ = .03). Finally, there was no association between autonomy regulation and external motivation (β = .03; *p* = .614), nor between agentic engagement and external motivation (β = -.03; *p* = .633; *R*^2^ = .00).

## Discussion

In Study 1, autonomy regulation was found to be strongly associated with intrinsic and internalized types of motivation. Moreover, the study revealed weak but positive correlations between autonomy regulation and introjected avoidance motivation and no significant correlations with external motivation. Speaking to the conceptual difference between intrapersonal and interpersonal autonomy regulation, agentic engagement only had positive associations with intrinsic motivation and was negatively associated with introjected avoidance motivation. Strikingly, although the measures used were based on distinct research paradigms (motivation regulation and SDT), autonomy need satisfaction and autonomy regulation were highly correlated. Earlier research showed that need satisfaction can mediate the effects of the self-regulation of needs on outcomes [10, 32]. Thus, there seems to be a conceptual and empirical difference between individuals’ feelings of autonomy and their strivings for autonomy.

## Study 2

We conducted a second study to improve some aspects of Study 1, namely, to compare whether the distinct strategies we identified in Study 1 can both be applied for interpersonal and intrapersonal autonomy regulation. To this end, we additionally introduced an interpersonal and an intrapersonal dilemma in which individuals need to regulate their autonomy. We referred to situations in which individuals feel they need to perform prescribed actions and assessed which self-supportive strategies individuals reported they would use to increase their autonomy.

Moreover, we assessed individuals’ overall tendency to create experiences centered around autonomy for themselves, to fulfill their need for autonomy by themselves. These general strivings for autonomy encompass strategies aimed at proactively creating autonomy-based experiences, which might also be associated with distinct types of motivation given the strong link between need satisfaction and motivation, and thus may play a significant role in shaping individuals’ motivations.

**Fig 2:**
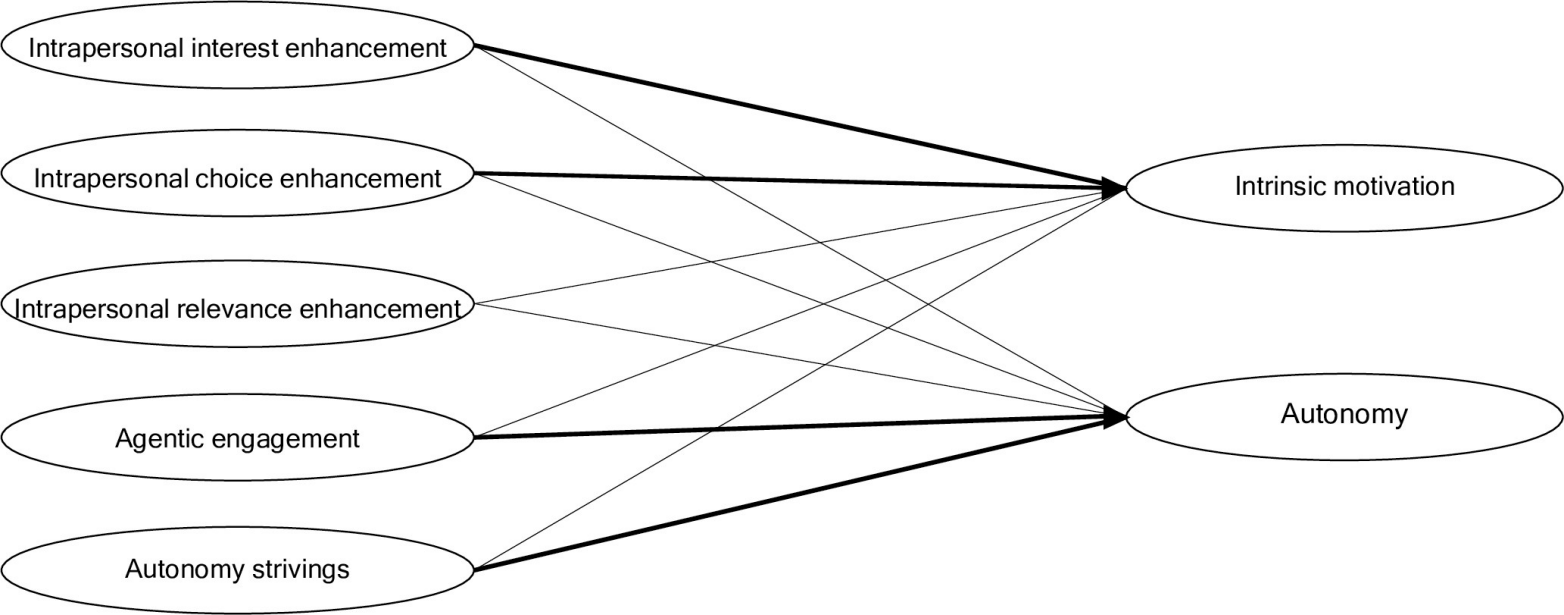
Associations between intrapersonal, interpersonal autonomy regulation and autonomy need satisfaction and motivation. *Note*. Strength of relationship is signalized through line thickness.

First, we expected the theoretical differentiation into general autonomy-enhancing strivings, intrapersonal autonomy regulation, and interpersonal autonomy regulation to hold empirically. Second, we expected to be able to differentiate between (sub-)facets of intra-personal, and interpersonal autonomy regulation concerning enhancing choices/self-determination, interest enhancement, and creating personal relevance. Third, we assumed that individuals with a higher endorsement of the different autonomy regulation strategies reported higher autonomy and intrinsic and identified motivation (i.e., positive correlations were expected, see Fig 2).

Fourth, we evaluated the convergent and divergent validity of the newly developed measures of autonomy regulation, by examining correlations between autonomy-regulating strategies and state emotions (feeling optimistic, isolated, lonely, bored, and stressed), state life satisfaction, and five HEXACO personality dimensions (i.e., emotionality, extraversion, agreeableness vs. anger, conscientiousness, and openness to experience; [66]).

Based on previous findings showing positive correlations between motivation regulation strategies and positive emotions [67] as well as (study) satisfaction [68], we expected positive associations of the distinct autonomy regulation strategies and individuals’ state optimism and life satisfaction. Concerning negative feelings, it is less straightforward to formulate hypotheses, because there are conflicting theoretical and empirical notions on the associations between motivation regulation strategies and negative states or feelings. Opelt and Schwinger ([67], Supplemental Material) report positive correlations between negative affect during learning (measured via the PANAS) with both enhancement of interest and personal relevance. Yet, the PANAS equates a variety of negative emotions [69], and therefore, this correlation might not inform about associations with specific emotional states. Boredom implies a lack of interest or intrinsic value concerning a task (e.g., [70]). Thus, boredom might be overcome by an enhancement of interest (see [71]), which may be reflected in a negative correlation between boredom and enhancement of interest. Moreover, previous findings confirmed a positive association between resilience and motivation regulation strategies [72]. According to Fried and Chapman [72], individuals using motivation regulation strategies might shape a greater sense of control over their experiences, resulting in greater resilience and stress resistance. Therefore, we assumed a negative association between autonomy regulation and perceived stress. Finally, considering SDT, loneliness, and isolation should be associated with relatedness-regulating strategies instead of autonomy-regulating strategies (e.g., [73]).

Prior research also confirmed correlations between motivation regulation and personality traits [74], particularly with conscientiousness [e.g., 75]. Accordingly, we expected positive associations between, on the one hand, openness to experience, extraversion, and conscientiousness, and, on the other hand, autonomy regulation strategies. Conversely, we expected negative correlations between autonomy regulation and emotionality (following [74]), as well as between autonomy regulation and agreeableness.

Concerning agreeableness, Ljubin-Golub et al. [74] confirmed positive associations with motivation regulation. Moreover, several studies investigating the associations between need satisfaction and personality traits found a positive correlation between autonomy need satisfaction and agreeableness in different contexts (e.g., [76, 77]; for a meta-analysis see [78]). One reason for the positive correlation could be a conceptual overlap of the autonomy measures with assisted autonomy strivings, e.g., through using measures that entail wordings that are similar to items such as “I feel like my surroundings allow me to express myself and my feelings” [31]. Specifically, Levine et al. [31] labeled assisted autonomy strivings next to agreeableness as collaborative personality factors that help individuals achieve greater need satisfaction and interpersonal support, due to their tendency to engage proactively with others when searching for autonomy. Individuals with high levels of agreeableness [79] and assisted autonomy [31] tend to perceive (and likely also receive) greater support from others. Therefore, it can be assumed that agreeableness and assisted autonomy strivings are positively correlated.

The prior measurement of motivation regulation did not take into account autonomy strivings or interpersonal autonomy regulation, which refers to being vocal or assertive about one’s needs or concerns when interacting with others. Interpersonal autonomy regulation can encompass asserted autonomy strivings, a tendency to claim personal autonomy and independence [29, 31].

Specifically, agentic engagement refers to advocating for one’s own needs and making oneself heard (exemplified in the saying “the squeaky wheel gets the grease," see [80]). Moreover, Young and colleagues [81] argue that agreeableness “may draw attention away from (…) own emotions, cognitions, and tasks” (p. 1333); consequently, individuals high in agreeableness can be characterized by directing their focus toward others instead of themselves, being socially attentive [82]. Therefore, a negative association between individuals’ agreeableness and their use of intrapersonal autonomy regulation strategies is also likely, e.g., because individuals high in agreeableness show a higher tendency to follow rules and are compliant with instructions [83].

### Method

#### Participants and procedure

Data for this study were collected in week 39, 2020. Again, people were invited to take part in the repeated cross-sectional branch of COSMO-Denmark [58]. This time, 8,000 people were invited, of whom 1,167 completed the survey. Among these participants, only a random subset saw the items pertaining to this study (i.e., approximately 50%). That is, the final sample for Study 2 consisted of 565 participants (45.57% male, 52.52% female, 0.17% other, *M*_*age*_ *=* 54.71, *SD =* 15.54 years) with active informed consent.

#### Measures

Following the same procedure as in Study 1, all scales were measured with a 5-point Likert scale ranging from 1 = *strongly disagree* to 5 = *strongly agree*, and items were translated from English into Danish. Intrinsic motivation, autonomy need satisfaction, and agentic engagement were measured with the same scales used in Study 1. The internal consistencies of the measures of autonomy regulation and motivation are reported in Table 4.

**Table 4:** Descriptive statistics, internal consistencies (Cronbach’s alpha), and intercorrelations of all variables (Study 2).

Latent factors CFA model	*M*	*SD*	α	(2)		(3)		(4)		(5)		(6)		(7)		(8)		(9)	
(1) Intrinsic motivation	3.02	0.95	.84	.30	***	.20	***	.38	***	.23	***	.20	***	.28	***	.17	***	.25	***
(2) Autonomy need satisfaction	3.84	0.48	.70			.80	***	.87	***	.59	***	.58	***	.49	***	.49	***	.61	***
(3) Agentic engagement	3.86	0.58	.74					.75	***	.59	***	.53	***	.45	***	.55	***	.61	***
(4) Autonomy strivings	3.68	0.55	.75							.68	***	.67	***	.64	***	.60	***	.74	***
(5) Intrapersonal choice enhancement	3.77	0.59	.59									.7	***	.90	***	.80	***		
(6) Intrapersonal relevance enhancement	3.79	0.59	.76											.72	***	.67	***		
(7) Intrapersonal interest enhancement	3.66	0.70	.74													.67	***		
(8) Interpersonal autonomy regulation	3.90	0.76	.89																
Latent factor higher order model																			
(9) Higher-order autonomy regulation	-	-	-																

*Note*. *N* = 558 (all measures).

*p < .05.

**p < .01.

***p < .001.

#### Autonomy strivings

Autonomy strivings were assessed with two items that assessed the self-support of creating choices that were adapted from the scale of Chen et al. [36]: “I look for a sense of choice and freedom in the things I undertake,” “I want my choices to express who I really am.” Four further newly developed items targeting general self-enhancement of interest and self-relevance (depicted in Table 5) were administered.

**Table 5:** Standardized factor loadings 6-factor CFA, Study 2.

		Theoretical dimensions					
		General autonomy regulation		Intrapersonal autonomy regulation			Interpersonal autonomy regulation
		Empirically differentiated factors					
Label	Item	Autonomy strivings	Agentic engagement	Choice enhancement	Interest enhancement	Relevance enhancement	Interpersonal autonomy regulation
AUEN1	I look for a sense of choice and freedom in the things I undertake.	.55					
AUEN2	I want my choices to express who I really am.	.55					
AUEN3	I try to make whatever I do as interesting as possible.	.66					
AUEN4	I try all kinds of different things to stimulate my interest.	.55					
AUEN5	I aim to create meaning for myself in the things I undertake.	.64					
AUEN6	I look for connections between my tasks and my life as such.	.50					
NRSR1	I tell others what I like and what I don’t like.		.63				
NRSR2	I express my preferences and opinions.		.73				
NRSR3	I offer suggestions about how to improve something if I do not like it.		.66				
NRSR4	When I need something, I’ll ask for it.		.59				
INTRAC1	If it is possible, I adjust the tasks to my preferences.			.61			
INTRAC2	I try to use the resources that I prefer to solve the problem.			.55			
INTRAC3	I aim to add variety to activities, such as tasks that I need to carry out repeatedly.			.58			
INTRAI1	I look for ways to bring more fun to the task.				.72		
INTRAI2	I try to combine a boring task with something pleasurable.				.59		
INTRAI3	I figure out a way to make the work more interesting.				.79		
INTRAR1	I tell myself that doing the task can be of great value to me later on.					0.60	
INTRAR2	I try to think about how completing the task will help me in my life.					0.68	
INTRAR3	I make myself focus on how the task helps me achieve my goals.					0.68	
INTRAR4	I consider a way to make tasks personally meaningful.					0.68	
INTERC1	I ask whether I can add variety to the task.						.72
INTERC2	I ask for more options and choices.						.74
INTERC3	I ask if I can perform the task in different ways.						.68
INTERI1	I ask for tasks that match my interests.						.62
INTERI2	I let others know which tasks I would like.						.60
INTERI3	I offer suggestions about how to make the task more interesting.						.74
INTERR1	I ask for tasks that I find personally relevant.						.65
INTERR2	I let others know which tasks I would find personally meaningful.						.69
INTERR3	I ask questions to help me understand the relevance of the task.						.59

*Note*. *N* = 558. CFA = Confirmatory factor analysis. All factor loadings were statistically significant (*p* < .001). Based on modification indices, correlated residuals were added between INTERR1 and INTERI1 (I ask for tasks that I find personally relevant; I ask for tasks that match my interests), between INTERC3 and INTERC1 (I ask whether I can add variety to the task; I ask if I can perform the task in different ways), between INTERI1and INTERI 2 (I ask for tasks that match my interests; I ask for tasks that match my interests), between INTERR2 and INTERI2 (I let others know which tasks I would find personally meaningful; I let others know which tasks I would like), and between AUEN1 and AUEN6 (I look for a sense of choice and freedom in the things I undertake; I look for connections between my tasks and my life as such).

#### Intra- and interpersonal autonomy regulation

Intrapersonal autonomy regulation was introduced with the dilemma: “Please think about situations in which you do not feel like working hard, or in which you do not feel like finishing a task, even when the work is not yet completed, and you know you should do more. Please indicate how frequently you use the following strategies in response to the situation: …” Interpersonal regulation was introduced with the dilemma: “Please think about situations in which you are assigned a task that you find irrelevant or boring. Please indicate how frequently you use the following strategies in response to the situation: …” Intra- and interpersonal autonomy regulation was assessed considering the autonomy-supportive strategies of stimulating interest, providing rationales, and providing choices [25]. The items are reported in Table 5; they were adapted from earlier research [11, 44, 45, 84, 85].

#### Measures used to examine convergent and divergent validity

Five emotional states were assessed with one-item measures on a 5-point Likert scale ranging from 1 = *not at all* to 5 = *extremely much*: “I am bored at the moment,” “I feel lonely at the moment,” “I feel isolated at the moment,” “I feel stressed at the moment,” “I am very optimistic when I think about the future.” Momentary life satisfaction was measured with the item “Overall, how satisfied are you with your life at the moment?”

The Brief HEXACO Inventory was implemented, assessing the six HEXACO personality dimensions with 4 items per dimension [86]. Five of the HEXACO personality dimensions were considered in the present study. The internal consistencies were low (emotionality: α = .34; extraversion: α = .59; agreeableness: α = .38; conscientiousness: α = .53; openness to experience: α = .54), which is a common finding concerning short measures of broad personality dimensions. Yet, prior research suggests that despite low reliability the measurement demonstrates sufficient concurrent validity [87]. A CFA showed acceptable model fit (CFI = .948; TLI = .916, RMSEA = .031; SRMR = .040), in case (1) the factor with the lowest reliability (emotionality) was excluded, and (2) correlated residuals were added, particularly amongst the residuals of all negatively formulated items. For the sake of completeness, we opted not to exclude emotionality from further analyses.

**Analyses.** We compared different parametrizations of autonomy regulation with CFAs and higher-order models (assessing different specifications for general autonomy strivings, context-oriented and task-oriented autonomy regulation). Although we had theoretical assumptions on the structure of our measure, which would favor confirmatory factor analyses, enabling us to model the specific latent factors we expected, we also employed a more exploratory approach estimating exploratory structural equation models (ESEM) to allow a comparison of results. We considered several fit criteria to assess model fit (RMSEA and SRMR < .08; CFI and TLI > .90, [62]), and inspected the AIC and BIC values for evaluating competing models [62–64]. SEMs with latent dependent variables (intrinsic motivation, autonomy need satisfaction) and latent predictor variables (autonomy regulation strategies) were estimated. We added correlated residuals between pairs of items, based on inspection of modification indices (see Notes of Table 5).

To assess convergent validity, we added the states (altogether) and personality dimensions (separately) to the final CFA models of autonomy regulation.

### Results

We estimated a CFA model with 8 latent factors, specifying agentic engagement, general autonomy strivings, and the 6 self-supportive intra- and interpersonal strategies of creating choices, enhancing interest, and self-relevance. The model fit was acceptable (CFI = 0.919; TLI = 0.907; RMSEA = .042; SRMR = .046), but several correlations, e.g., between the interpersonal strategies enhancing interest and self-relevance, were above 1. This problem also arose in a model with 7 latent factors between the interpersonal strategies enhancing interest/self-relevance and choices. Therefore, these three interpersonal strategies were modeled as one latent factor. Because the latent factors explained too little variance in the respective indicators, two items were eliminated (“I try to find my own way to approach a task,” “I make the work more enjoyable by turning it into a game”). Based on similar item wordings and resulting significant modification indices, five pairs of correlated residuals were added (see Table 5). The CFAs resulted in a clearer factor interpretation than ESEM (see https://osf.io/96dm7). Moreover, the more latent factors we added, the more factor loadings above 1 we found, which is a sign of improper solutions or could also signalize over-fitting, which is a risk when testing ESEM:”a large number of freely estimated parameters can lead to over-fitting” ([88], p. 548). Therefore, we opted for the more parsimonious solution and only report the CFA results in the manuscript.

A CFA model with 6 latent factors yielded acceptable model fit and was superior to several other model specifications (see Table 6 for models and fit indices).

**Table 6:** Model fit of CFA models, Study 2.

Model	χ^2^	SCF	df	AIC	BIC	CFI	TLI	RMSEA	SRMR
Model comparison autonomy regulation strategies									
**6-factor CFA**	**699.53**	**1.30**	**357**	**39204.06**	**39666.76**	**0.925**	**0.915**	**0.041**	**0.047**
4-factor CFA	784.29	1.30	366	39298.81	39722.594	0.909	0.899	0.045	0.049
3-factor CFA (3 factors: general, intra- and interpersonal autonomy regulation)	853.19	1.31	369	39386.53	39797.35	0.894	0.884	0.048	0.051
3-factor CFA model (3 factors: autonomy strivings, agentic engagement, overall intra- and interpersonal autonomy regulation)	989.34	1.31	369	39563.78	39974.60	0.865	0.851	0.055	0.056

*Note*. *N* = 558. CFA = Confirmatory factor analysis. SCF = Scaling Correction Factor. df = degrees of freedom. AIC = Akaike information criterion. BIC = Bayesian information criterion. CFI = Comparative fit index. TLI = Tucker-Lewis index. RMSEA = Root mean square error of approximation. SRMR = Standardized root mean square residual. The selected models are highlighted in bold. Models with 7 or 8 latent factors yielded correlations above 1 between aspects of interpersonal autonomy regulation. Therefore, all interpersonal strategies were merged into 1 latent factor.

All factor loadings were above .50 (see Table 5).

Subsequently, we tested a model in which a higher-order factor was modeled to underlie the newly created intra- and interpersonal strategies (CFI = 0.926; TLI = 0.918; RMSEA = .038; SRMR = .047). Concerning the higher-order factor, all factor loadings were high (intrapersonal choice enhancement: .97; intrapersonal interest enhancement: .86; intrapersonal relevance enhancement: .84; interpersonal autonomy regulation: .81). The correlations between the higher-order autonomy regulation factor and agentic engagement (*r* = .60; *p* < .001) or with autonomy strivings (*r* = .74; *p* < .001) were lower. Moreover, given that a higher-order model in which these two aspects were specified to load on the higher-order factor alongside the other strategies had a comparably lower model fit (CFI = 0.900; TLI = 0.891; RMSEA = .044; SRMR = .056), the two factors were considered as separate factors.

The descriptive statistics, internal consistencies, and intercorrelations of all variables are shown in Table 4. The global index of intra- and interpersonal strategies had higher correlations with autonomy need satisfaction (*r* = .61; *p* < .001) than with intrinsic motivation (*r* = .25; *p* < .001). General autonomy strivings had the highest positive correlations with intrinsic motivation (*r* = .38; *p* < .001) and with autonomy need satisfaction (*r* = .87; *p* < .001) compared to the other constructs correlating with intrinsic motivation and autonomy need satisfaction.

Therefore, we tested the separability of autonomy need satisfaction, general autonomy strivings, and agentic engagement through comparing models with 1 factor (on which all items of autonomy need satisfaction, autonomy strivings, and agentic engagement loaded, CFI = 0.906; TLI = 0.882; RMSEA = .066; SRMR = .049) and 3 factors (CFI = 0.953; TLI = 0.937; RMSEA = .048; SRMR = .039). We added correlated residuals for two parallel items. The 3-factor model was superior to the 1-factor model. We also tested a 2-factor model in which autonomy need satisfaction and autonomy strivings (which had been measured with similar items) were specified as one factor next to agentic engagement (CFI = 0.937; TLI = 0.920; RMSEA = .055; SRMR = .043), which had marginally lower model fit. Based on these results and in line with the theoretical expectations, autonomy need satisfaction, autonomy strivings, and agentic engagement were modelled as three separate factors.

Due to the high intercorrelations, the higher-order factor model was used in the SEM models. General autonomy strivings were more strongly positively related with intrinsic motivation and autonomy than the global factor representing intra- and interpersonal strategies as a response to dilemmas. More precisely, the higher-order factor of autonomy regulation, representing a global index of intra- and interpersonal-oriented autonomy regulation, did not have statistically significant effects on intrinsic motivation and autonomy need satisfaction when general autonomy strivings and agentic engagement were considered (see Table 7).

**Table 7:** Predictive effects of aspects of autonomy regulation on intrinsic motivation and autonomy need satisfaction (Study 2).

	Intrinsic motivation			Autonomy need satisfaction		
Predictors	β	(SE)	*p*	β	(SE)	*p*
Autonomy strivings	.56	.16	.000	.69	.13	.000
Agentic engagement	-.18	.11	.089	.35	.10	.001
Global autonomy regulation	-.05	.11	.653	-.11	.08	.201
*R* ^2^	.16	.05	.001	.81	.06	.000

*Note*. *N* = 558.β = standardized regression coefficient. SE = Standard error. Results of two SEM models in which 2 latent factors of general autonomy regulation (autonomy strivings and agentic engagement) and 1 higher-order factor of global autonomy regulation (on which all aspects of task-oriented autonomy regulation and context-oriented autonomy regulation loaded) were modelled as predictors of the latent factors of intrinsic motivation and autonomy need satisfaction, respectively.

The results also showed the close correspondence of autonomy strivings and autonomy need satisfaction concerning the high amount of variance explained (*R*^2^ = .81). The results of the examination of the convergent and divergent validity of the autonomy-regulation strategies are reported in Table 8 (which also depicts the descriptive statistics of the additional state and trait measures).

**Table 8:** Descriptive statistics and intercorrelations of the convergent validity measures and autonomy regulation strategies (Study 2).

			Autonomy strivings		Agentic engagement		Intrapersonal choice enhancement		Intrapersonal interest enhancement		Intrapersonal relevance enhancement		Interpersonal autonomy regulation	
	*M*	*SD*	*r*	*p*	*r*	*p*	*r*	*p*	*r*	*p*	*r*	*p*	*r*	*p*
States														
Bored	2.03	1.10	-0.02	.662	-0.06	.190	-0.02	.704	-0.04	.427	-0.07	.193	-0.02	.739
Lonely	1.99	1.13	0.00	.960	-0.09	.095	0.02	.781	-0.07	.187	-0.05	.333	-0.06	.228
Isolated	2.29	1.19	0.01	.819	-0.06	.254	0.02	.724	-0.03	.519	-0.01	.825	0.02	.735
Optimistic	3.41	0.91	0.07	.190	**0.13**	**.025**	-0.02	.756	0.03	.527	0.05	.341	**0.13**	**.020**
Satisfied	3.67	0.88	0.01	.791	**0.13**	**.027**	0.05	.460	0.06	.237	0.06	.263	0.09	.114
Stressed	2.33	1.22	0.00	.973	-0.09	.092	-0.08	.147	**-0.11**	**.032**	-0.05	.325	-0.02	.622
Traits														
Conscientiousness	3.71	0.68	**0.22**	**.001**	**0.21**	**.001**	0.04	.605	**0.17**	**.019**	**0.34**	**.000**	0.08	.219
Openness	3.59	0.73	**0.44**	**.000**	**0.39**	**.000**	**0.37**	**.000**	**0.26**	**.000**	**0.26**	**.000**	**0.34**	**.000**
Agreeableness	3.28	0.63	**-0.14**	**.024**	**-0.41**	**.000**	**-0.27**	**.001**	**-0.14**	**.022**	**-0.17**	**.005**	**-0.29**	**.000**
Extraversion	4.10	0.66	**0.37**	**.000**	**0.49**	**.000**	**0.29**	**.000**	**0.31**	**.000**	**0.30**	**.000**	**0.31**	**.000**
Emotionality	2.98	0.68	0.08	.532	**-0.30**	**.015**	0.12	.360	0.12	.329	0.19	.085	0.10	.419

*Note*. The statistically significant correlations are highlighted in bold. For completeness reasons, we also calculated the correlations with emotionality, yet, this construct had the lowest reliability, and sufficient validity (via CFA) was only confirmed without emotionality.

The model fits were all acceptable, also when emotionality was considered (see https://osf.io/96dm7). We found statistically significant positive correlations between the overall factor of interpersonal autonomy regulation and state optimism, as well as between agentic engagement and state optimism. Moreover, we found a statistically significant positive correlation between agentic engagement and state satisfaction.

We could not confirm statistically significant associations between any autonomy-regulating strategy and boredom, loneliness, or isolation. We found a statistically significant negative correlation between intrapersonal interest enhancement and state stress.

Concerning personality dimensions, conscientiousness showed a statistically significant positive correlation with autonomy regulation strategies, except intrapersonal choice enhancement and context-oriented autonomy regulation. Openness to experience and extraversion showed a statistically significant positive correlation with all autonomy regulation strategies. Agreeableness showed statistically significant negative correlations with all autonomy regulation strategies. Emotionality showed a statistically significant negative correlation with agentic engagement. In our sample, measuring autonomy need satisfaction as self-congruence with choices and actions (which can be assumed to have lower correspondence with assisted autonomy strivings), the correlation between autonomy need satisfaction and agreeableness was *r* = -0.023; *p* = .826. These results mirrored findings by Weinstein et al. [21] who also found no correlations between three aspects of autonomy need satisfaction (self-congruence, susceptibility to control, interest-taking in own autonomy) and agreeableness.

## Discussion

Study 2 confirmed that individuals apply autonomy regulation when dealing with specific dilemmas. Moreover, the expected associations between autonomy regulation and the fulfillment of autonomy and intrinsic motivation in an important life domain were found. Yet, the self-supportive strategies in response to specific problems, such as finding the motivation to engage in or finish a task (intrapersonal dilemma), or to actively ask for a change in tasks (interpersonal dilemma), were found to have weaker associations with intrinsic motivation and autonomy need satisfaction than general strivings for autonomy.

## General discussion

When people cannot find reasons why an uninteresting task is meaningful, their autonomy—the need to act in ways that are meaningful for oneself—is impeded. However, if individuals encounter a motivational problem, e.g., when they have to complete a boring task, they can actively strive to improve their motivation (e.g., [44]). Yet, little is known about which type of motivation (intrinsic, identified, introjected avoidance or approach regulation, or external motivation) individuals can achieve through applying motivation regulation. There is a dearth of research on the associations of motivation regulation strategies with introjected or identified motivation. This is critical given that intrinsic motivation has been revealed to be an underlying factor to well-being in distinct life domains [7, 8] and identified motivation has been shown to drive persistence [7] and work performance [8], amongst other positive outcomes, whereas introjected and external motivation have been shown to have well-being costs (e.g., [8]).

In two studies, we provided evidence that individuals report using a set of self-regulatory strategies to create greater autonomy for themselves, through applying self-supportive strategies. Testing our first objective, it was confirmed in the two studies that the distinction of autonomy regulation strategies holds empirically.

Our second objective targeted the association of autonomy regulation strategies with perceived autonomy need satisfaction, intrinsic motivation, and distinct types of extrinsic motivation. In both studies, substantial associations of autonomy regulation with intrinsic motivation, identified motivation, and autonomy were found.

In Study 1, we found that autonomy regulation has stronger associations with autonomy need satisfaction, intrinsic motivation, identified motivation, and introjected approach motivation than with introjected avoidance motivation and external motivation (e.g., [6]). The associations revealed in Study 1 mirror SDT’s assumptions that relative autonomy is associated with extrinsic and intrinsic types of motivation [6]. Extrinsically motivated actions are assumed to come into play when autonomy is thwarted or hard to achieve, and accordingly, external motivation was not statistically significantly associated with autonomy regulation in Study 1. By contrast, behaviors regulated by introjected, identified, or intrinsic motivation should reflect increasing or complete autonomy, and, indeed, introjected avoidance motivation was weakly correlated with autonomy regulation, whereas identified and intrinsic motivation were substantially correlated with autonomy regulation. Thus, Study 1 highlighted that the quality of motivation (extrinsic versus intrinsic) is associated with the relative autonomy individuals achieve to create for themselves.

Study 2 yielded additional evidence that individuals can use autonomy regulation generally, which was found to be associated with their autonomy need satisfaction and intrinsic motivation in personally important life domains, in addition to associations with the self-supportive strategies they apply to tackle specific autonomy-related problems induced by the task or context with intra- and interpersonal autonomy regulation. When evaluating convergent validity, concerning personality dimensions, the patterns in the correlations largely confirm our theoretical assumptions. For example, agreeableness was found to be negatively associated with all autonomy regulation strategies. This could mean that our measures had a closer correspondence with asserted autonomy strivings. It would be interesting to develop new instruments that capture assisted autonomy strivings and assess the degree to which individuals strive for autonomy with social attentiveness and an eye for cooperation [81–83]. The correlations we found for intrapersonal autonomy regulation show that the nuances of claiming independence might also be associated with specific strategies, e.g., we found higher negative correlations between agreeableness and creating choices than with interest or relevance enhancement.

Concerning the association of autonomy regulation with momentary states, we found only a few correlations (although their directions align with theoretical expectations, [67, 68]). Notably, contrary to our assumption, we did not find statistically significant correlations between autonomy regulation strategies and state boredom. Being bored typically means one is not intrinsically motivated. Yet, autonomy regulation might be a means to change how one (dis-)engages in a situation or with a task, through creating a sense of ownership and making contexts or tasks more inherently interesting. Therefore, our finding contradicts theoretical explanations [70, 71] and findings, e.g., by Sansone et al., [51] showing that individuals know various ways to make uninteresting tasks more intrinsically motivating. For example, an uninteresting duty can be reframed as an artistic task, the environment can be changed, or the procedure can be varied [51]. Particularly varying the procedure was shown to be a widely used strategy as a means to increase interest in an uninteresting task ([51], Table 2).

The reason why we could not find correlations with states but corroborate our theoretical assumptions concerning the traits might be measurement misalignment. It is possible that deeper insights into the associations between autonomy regulation and feelings could be gained by examining momentary feelings and immediate responses in autonomy regulation.

In both studies, the associations among autonomy need satisfaction, agentic engagement, and autonomy regulation were high. In Study 2, agentic engagement, general autonomy strivings, and autonomy need satisfaction were shown to be three distinct, yet highly interrelated different constructs. Agentic engagement had higher correlations with autonomy need satisfaction and general autonomy strivings than with intra- and interpersonal autonomy regulation strategies. Likewise, general autonomy strivings were revealed to have higher correlations with autonomy need satisfaction than with strategies that were applied as intra- or interpersonal strategies. Thus, agentic engagement and autonomy strivings seemed to express a different self-supportive style than the intra- and interpersonal strategies in response to a specific problem and to reflect strategies that people apply to navigate towards greater autonomy fulfillment across distinct situations and environments.

### Limitations

Some limitations need to be considered when interpreting the results of the present research. First, our studies were conducted in one country via self-report and had a cross-sectional design. Due to its cross-sectional nature, our study cannot offer insights into the dynamics at play concerning the impact of motivation regulation on the quality of individuals’ motivation. Hence, the findings are limited by correlational methods. The generalizability of this research’s findings should be investigated in other samples to investigate whether the autonomy regulation strategies we found can be considered as universally applied strategies.

Second, we used domain-general items for motivation, autonomy need satisfaction, and autonomy regulation. Although we introduced two dilemmas in Study 2, these were hypothetical. Thus, it might be fruitful for future research to assess distinct autonomy regulation strategies while people are working on a specific task that needs to be completed and has an impact on personally important outcomes, in order to assess the impact of the use of general and context-oriented autonomy strivings in detail.

Third, because we wanted to capitalize on existing measures of motivation regulation and agentic engagement in order to integrate the findings of distinct research paradigms, we only focused on three autonomy-supportive strategies (creation of choices, enhancement of self-relevance, and interest). Yet, further autonomy regulation strategies, such as informational self-talk (based on the autonomy-supportive strategy informational language, see [22]) should be considered, too. Likewise, we only focused on the self-regulation of autonomy, while people’s self-regulating actions might also be driven by their needs for competence and relatedness [e.g., 4] or other psychological needs.

Fourth, it needs to be noted that in higher-order models, the associations between the higher-order factor and the manifest items are defined by the associations between both the higher- and lower-order factors and the lower-order factors and the item. As a consequence, the associations between the higher-order factor and items are constrained.

Fifth, the model in which autonomy need satisfaction and autonomy strivings were differentiated versus the model in which autonomy need satisfaction and autonomy strivings were modeled as one factor only was marginally superior. Based on our theoretical conceptualization, we still opted for the distinction, but future research should test further measures that allow disentangling strategies that aim to proactively induce autonomy-based experiences from autonomy need satisfaction. Correspondingly, the non-significant regression coefficients might also imply multicollinearity due to the substantial correlations between the predictors, such as between autonomy need satisfaction and autonomy strivings. Therefore, future research should draw on experimental or longitudinal designs, in order to yield further insights into how feelings and strivings of autonomy can be distinguished.

Sixth, the reliability of the personality measures was low. Nevertheless, several studies confirmed the concurrent validity of the personality measures [86, 87], as well as their predictive validity [89]. Moreover, the model fits of the CFAs were adequate, and CFAs and SEMs allow for correcting for measurement error [90], which underlines that conducting CFAs and SEMs was the appropriate strategy in our analyses.

### Implications

Our studies have important theoretical and practical implications. First, our findings highlight the conceptual overlap of seemingly divergent operationalizations from different theoretical paradigms. The idea that persons can regulate their own motivation has been highlighted in different theoretical frameworks, such as the paradigm of self-regulated learning (SRL, e.g., [27]), or the paradigm of academic engagement [39]. Moreover, a boring or irrelevant task can be claimed to thwart a person’s basic psychological need for autonomy (e.g., [1, 36]). Therefore, we used the framework of SDT to contextualize motivational regulation strategies as distinct self-supportive strategies that induce feelings of autonomy (e.g., [32]). Items that could be traced back to distinct paradigms and for which one could argue that they target distinct constructs (motivation regulation or self-support of autonomy) seem to measure and reflect the same underlying regulation strategy. Our research focused on the conceptual similarities of motivation regulation and autonomy regulation, and future research should explore potential differences of these constructs or derive a comprehensive overview of their similarities.

Second, notably, when facing unpleasant activities in their daily life, individuals’ spontaneous reaction seems to concentrate on external motivation (focusing on positive consequences of activities [24]). Several studies have shown that contextual autonomy support can facilitate the process of internalization of extrinsic reasons (turning them into intrinsic reasons; [9, 43]). However, little is known about how more controlled forms of motivation can become more autonomously regulated using self-supportive strategies. Considering the associations of autonomy regulation with positively-valenced outcomes, such as identified motivation, intrinsic motivation, and autonomy need satisfaction, it is relevant to investigate how autonomy regulation can be fostered and how the promotion of autonomy regulation affects individuals’ motivation (see also [10]).

Given the evidence on the effectiveness of an autonomy-supportive intervention in boosting agentic engagement and the self-generation of autonomy need satisfaction (e.g., [91]), it seems promising to implement autonomy-supportive interventions. For example, it might be fruitful to design intervention studies priming the use of intrapersonal and interpersonal strategies. This would allow for examining how individuals with generally high autonomy strivings regulate their autonomy need satisfaction and motivation in these circumstances.
